# Highly Emissive Blue Quantum Dots with Superior Thermal Stability via In Situ Surface Reconstruction of Mixed CsPbBr_3_–Cs_4_PbBr_6_ Nanocrystals

**DOI:** 10.1002/advs.202104660

**Published:** 2021-12-26

**Authors:** Hyeonjung Kim, Jong Hyun Park, Kangyong Kim, Dongryeol Lee, Myoung Hoon Song, Jongnam Park

**Affiliations:** ^1^ School of Energy and Chemical Engineering Ulsan National Institute of Science and Technology (UNIST) UNIST‐gil 50 Ulsan 44919 Republic of Korea; ^2^ Department of Materials Science and Engineering Ulsan National Institute of Science and Technology (UNIST) UNIST‐gil 50 Ulsan 44919 Republic of Korea; ^3^ Department of Biomedical Engineering Ulsan National Institute of Science and Technology (UNIST) UNIST‐gil 50 Ulsan 44919 Republic of Korea

**Keywords:** blue quantum dots, light‐emitting diodes, perovskites, surface reconstruction, thermal stability

## Abstract

Although metal halide perovskites are candidate high‐performance light‐emitting diode (LED) materials, blue perovskite LEDs are problematic: mixed‐halide materials are susceptible to phase segregation and bromide‐based perovskite quantum dots (QDs) have low stability. Herein, a novel strategy for highly efficient, stable cesium lead bromide (CsPbBr_3_) QDs via in situ surface reconstruction of CsPbBr_3_–Cs_4_PbBr_6_ nanocrystals (NCs) is reported. By controlling precursor reactivity, the ratio of CsPbBr_3_ to Cs_4_PbBr_6_ NCs is successfully modulated. A high photoluminescence quantum yield (PLQY) of >90% at 470 nm is obtained because octahedron CsPbBr_3_ QD surface defects are removed by the Cs_4_PbBr_6_ NCs. The defect‐engineered QDs exhibit high colloidal stability, retaining >90% of their initial PLQY after >120 days of ambient storage. Furthermore, thermal stability is demonstrated by a lack of heat‐induced aggregation at 120 °C. Blue LEDs fabricated from CsPbBr_3_ QDs with reconstructed surfaces exhibit a maximum external quantum efficiency of 4.65% at 480 nm and excellent spectral stability.

## Graphical abstract

Highly emissive blue cesium lead bromide (CsPbBr_3_) quantum dots are developed by controlling the reactivity of metal precursors. In situ generated Cs_4_PbBr_6_ nanocrystals eliminate the surface defects of CsPbBr_3_ quantum dots, resulting in the high colloidal and thermal stability. Fabricated blue light‐emitting diode shows external quantum efficiency of 4.65% at 480 nm and excellent spectral stability under operational conditions.

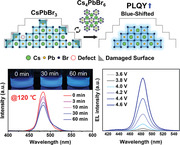

## 1 Introduction

Metal halide perovskite materials have been recognized as promising candidates for next‐generation color displays because their high photoluminescence quantum yields (PLQYs), narrow full widths at half maximum (FWHM), ease of bandgap tuning, and solution processability fulfil the ITU‐R Recommendation BT.2020 (Rec. 2020) of the International Telecommunication Union (ITU).^[^ ^1^, ^2^, ^3^ ^]^ Recently, significant progress has been achieved in the development of near‐infrared, red, and green perovskite light‐emitting diodes (LEDs), with external quantum efficiencies (EQEs) reaching over 20%.^[^ ^4^, ^5^, ^6^, ^7^, ^8^ ^]^ However, the efficiency of blue perovskite materials has lagged far behind, with EQEs of 12.3% in the sky blue region of the spectrum (475–490 nm) and 8.8% in the blue region (460–475 nm) having been reported.^[^ ^9^, ^10^ ^]^ Furthermore, Joule heating during LED operation is inevitable; thus, the development of perovskite materials with superior thermal stability, in which thermal quenching is minimized, is an important issue for the practical application of perovskite LEDs.

Several strategies for obtaining blue‐emitting perovskites nanocrystals (NCs) are available. One method involves mixed halides that include both Br and Cl anions.^[^ ^11^, ^12^, ^13^, ^14^, ^15^, ^16^, ^17^ ^]^ Although this is a convenient method for bandgap engineering, easy formation of Cl^−^ vacancies poses a limitation as it results in a deep trap state within the bandgap.^[^ ^18^, ^19^, ^20^ ^]^ These defect sites cause perovskite layer degradation and ion migration, resulting in phase segregation in response to the application of an electric field during device operation.^[^ ^21^, ^22^ ^]^ A second method is to use Br‐based 2D perovskite nanoplatelets and take advantage of the exciton quantum confinement effect.^[^ ^23^, ^24^, ^25^, ^26^, ^27^ ^]^ In inorganic cesium lead bromide (CsPbBr_3_) nanoplatelets, the emission can be controlled according to the number of [PbBr_6_]^4−^ layers; however, strong exciton–phonon coupling and a randomly oriented distribution of nanoplatelets result in low‐performance LEDs.^[^ ^28^ ^]^ A third strategy for achieving blue‐emitting perovskites NCs is to reduce the crystal size of a perovskite material such that it is within the quantum confinement regime.^[^ ^9^, ^29^, ^30^, ^31^, ^32^ ^]^ CsPbBr_3_ NCs with sizes in the quantum confinement regime (denoted by quantum dots, QDs) usually suffer from low PLQYs and stability because they are strongly affected by surface defects when the surface‐to‐volume ratio is high.^[^ ^33^ ^]^ In particular, small‐sized QDs are easily degraded and undergo aggregation because of their high surface energy, leading to broad emission spectra and poor spectral stability such that the emission color is susceptible to changing to green at high temperature. To overcome these problems, various approaches have been attempted, including amorphous CsPbBr*_x_* shelling,^[^ ^31^ ^]^ the addition of excess Br using ZnBr_2_ as the source,^[^ ^29^ ^]^ Sb^3+^ doping,^[^ ^32^ ^]^ and acid etching‐driven ligand exchange.^[^ ^34^ ^]^ However, few studies have been conducted on the application of blue LEDs incorporating highly stable CsPbBr_3_ QDs.^[^ ^9^, ^34^ ^]^ A quite different approach involves CsPbBr_3_ NCs being embedded in a Cs_4_PbBr_6_ matrix with a crystal size of several hundred nanometers or more, resulting in improved PLQY and stability.^[^ ^35^, ^36^, ^37^, ^38^ ^]^ However, the literature reports in this area have been focused only on green emission, and Cs_4_PbBr_6_/CsPbBr_3_ embedded structures are not suitable for electroluminescent devices because a 0D Cs_4_PbBr_6_ phase has a wide bandgap of 3.95 eV and is insulating.^[^ ^39^, ^40^ ^]^

In this article, we report a unique method to enhance the PLQY and stability of blue‐emitting CsPbBr_3_ QDs via simultaneous generation of mixed CsPbBr_3_ QDs and Cs_4_PbBr_6_ NCs. By controlling the reactivity of the precursors, the size of the CsPbBr_3_ QDs was controlled. As a result, an emission wavelength of 470 nm with a high PLQY of >90% was achieved. We investigated the effects of the Cs_4_PbBr_6_ NCs on the photophysical properties and thermal stability of the CsPbBr_3_ QDs by observing changes in their morphology and optoelectronic properties. Octahedron defect sites on the surface of CsPbBr_3_ QDs were etched by the Cs_4_PbBr_6_ NCs, resulting in defect removal. This CsPbBr_3_ QD surface reconstruction decreases the defect density and eliminates nonradiative recombination pathways, leading to high efficiency and stability for the CsPbBr_3_ QDs. The mixed NC solution retained 90% of its initial PLQY value over 120 days of storage under ambient conditions, with little change in the emission peak position and FWHM. Thermally induced aggregation and fusion were suppressed during 60 min of heating at 120 °C. Spectrally stable and efficient blue LEDs, having an EQE of 4.65% at 480 nm, based on the mixed NCs were achieved.

## 2 Results and Discussion

### 2.1 Preparation of In Situ Generated Blue‐Emitting NCs

**Figure 1:**
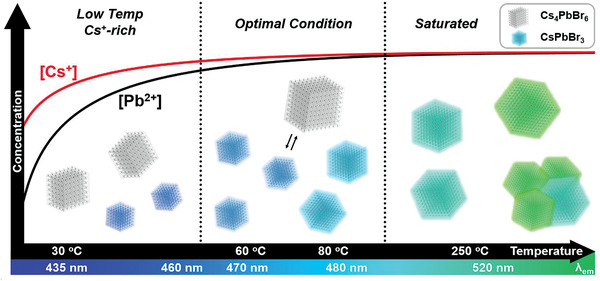
Schematic representation of temperature‐dependent growth trend of perovskite nanoparticles. The schematic depicts the active metal precursor ion concentrations and products (CsPbBr_3_ and Cs_4_PbBr_6_ nanocrystals) as functions of increasing reaction temperature. The colored *x*‐axis that gradually changes from blue to green indicates the CsPbBr_3_ NCs emission wavelength.

We synthesized a mixed solution of CsPbBr_3_ and Cs_4_PbBr_6_ NCs by modifying a previously reported synthetic method (details provided in the Experimental Section).^[^ ^41^ ^]^ In a typical synthesis, cesium carbonate (Cs_2_CO_3_), lead oxide (PbO), oleic acid (OA), and 1‐octadecene (ODE) were added to a three‐necked round‐bottom flask, and metal oleate complexes were formed by heating at 120 °C. Oleylammonium bromide (OLAM‐Br) was prepared separately by reacting oleylamine and hydrobromic acid (HBr), and then this was injected at low temperature under Ar into the metal oleate complexes. In this step, the reaction temperature and the Cs to Pb precursor ratio, as important factors for obtaining high‐quality NCs with blue emission in the range of 460–480 nm, were carefully controlled. The CsPbBr_3_ to Cs_4_PbBr_6_ NC formation ratio was modulated by varying the Cs to Pb precursor feed ratio, as described in a previous report.^[^ ^42^ ^]^ When the amount of Pb precursor exceeded the amount of Cs precursor, the formation of CsPbBr_3_ NCs in the orthorhombic phase was favored (Figure S1, Supporting Information). When the Cs and Pb precursor ratio was fixed at 1:1 and the reaction temperature was reduced, the growth of NCs was suppressed, and small CsPbBr_3_ NCs and Cs_4_PbBr_6_ NCs were simultaneously co‐synthesized. The emission wavelength of the QDs was 470 nm, and a high PLQY of above 90% was achieved. This phenomenon occurred because the depletion of the Pb precursor was slower than that of the Cs precursor.^[^ ^42^, ^43^ ^]^ At low temperature, the difference between the reactivities of the metal precursors was maximized, hence formation of Cs_4_PbBr_6_ NCs was promoted and the size of the CsPbBr_3_ NCs was reduced (**Figure** **1**).

**Figure 2:**
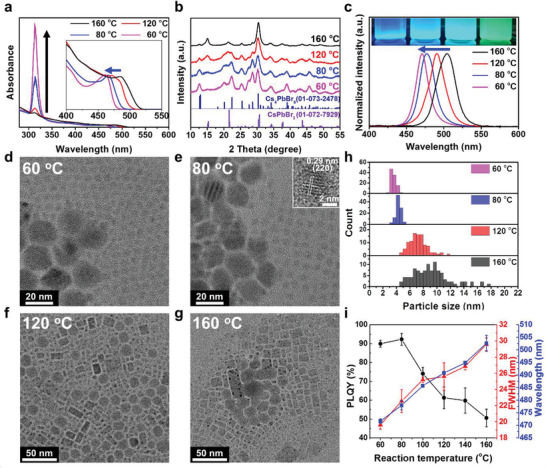
Characterization of perovskite NCs depending on the reaction temperature. a) UV–vis absorption spectra, b) XRD patterns, and c) PL spectra of samples synthesized at 60, 80, 120, and 160 °C, respectively. The insets are photographs of perovskite NCs in nonpolar solvents acquired under 365 nm UV light illumination. TEM images of the samples synthesized at d) 60 °C, e) 80 °C, f) 120 °C, and g) 160 °C; the inset of (e) shows a high‐resolution TEM image the of CsPbBr_3_ NCs prepared at 80 °C. h) Histograms illustrating the distributions of CsPbBr_3_ NC sizes for the samples prepared at different reaction temperatures. i) Variations in emission wavelength, PLQY, and FWHM of the as‐synthesized products with reaction temperature.

We investigated the dependence of the photophysical properties of the as‐synthesized NCs on the reaction temperature in the range of 60–160 °C. These NCs exhibited two peaks in the UV–vis absorption spectra: the first excitonic peak, between 450 and 500 nm, and a sharp peak centered at 313 nm (**Figure** **2**a). The absorption peak at 313 nm originated from optical transitions between localized states of the isolated [PbBr_6_]^4−^ octahedron of Cs_4_PbBr_6_ NCs.^[^ ^44^, ^45^, ^46^ ^]^ X‐ray powder diffraction (XRD) patterns of the NCs allowed us to confirm that the first exciton peak is related to the bandgap of the luminescent CsPbBr_3_ NCs. As shown in Figure 2b, the sample of synthesized NCs included both orthorhombic CsPbBr_3_ NCs and rhombohedral Cs_4_PbBr_6_ NCs, and the Cs_4_PbBr_6_‐to‐CsPbBr_3_ ratio increased as the reaction temperature decreased. This result is consistent with the increase in the intensity of the absorption peaks at 313 nm in the UV–vis spectra. As the reaction temperature decreased from 160 to 60 °C, the first exciton peak was blueshifted, and the emission peak center shifted from the green (504 nm) to the blue (470 nm) wavelength region (Figure 2c). Transmission electron microscopy (TEM) analysis also indicated that CsPbBr_3_ and Cs_4_PbBr_6_ NCs coexisted in the samples, and lowering the temperature reduced the overall size of the NCs (Figure 2d–g and Figure S2 (Supporting Information)). In addition, when the particle sizes (diameters) were measured by focusing on the luminescent CsPbBr_3_ NCs, the average size and relative standard deviations decreased from 9.3 to 3.5 nm and from 38.1% to 9.0%, respectively, as the reaction temperature decreased from 160 to 60 °C, because of the slow crystal growth rate at low temperature (Figure 2h). Because the size of the CsPbBr_3_ NCs synthesized at a temperature below 160 °C was smaller than the exciton Bohr radius (≈7 nm),^[^ ^47^ ^]^ the blueshift in the emission peaks resulted from a quantum confinement effect, and the reduction in FWHM was due to the size distribution and defect density decrease. It should be noted that as the reaction temperature decreases, the PLQY increases from 57.7% to 90.1%, contrary to previously reported general trends of increasing crystallinity and PLQY with temperature (Figure 2i).^[^ ^41^ ^]^

### 2.2 Stability of In Situ Generated Perovskite NCs

**Figure 3:**
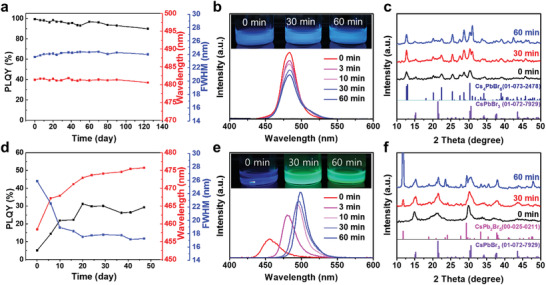
Stability of in situ generated perovskite NCs. Variations in emission wavelength, PLQY, and FWHM of a) in situ generated CsPbBr_3_–Cs_4_PbBr_6_ NCs (ISNCs) and d) conventional CsPbBr_3_ QDs (C‐QD_113_) with incubation time under ambient conditions (relative humidity: 65%, temperature: 25 °C). Emission stability of b) ISNCs and e) C‐QD_113_ in toluene at 120 °C; the insets show photographs of the respective NCs acquired under UV light illumination. XRD patterns of c) ISNCs and f) C‐QD_113_ after annealing in toluene at 120 °C over various periods of time.

In order to analyze the stability of the in situ generated CsPbBr_3_–Cs_4_PbBr_6_ NCs (denoted by ISNCs), we synthesized small CsPbBr_3_ QDs (denoted by C‐QD_113_) as a control group using a conventional method based on hot injection of Cs‐oleate at 90 °C with control of the OA and oleylamine (OLAM) ligands (details provided in the Figure S3 in the Supporting Information and the Experimental Section).^[^ ^48^ ^]^ Conventional CsPbBr_3_ QDs (denoted by C‐QD_113_) were obtained by hot injection of Cs‐oleate at 90 °C with control of the OA and OLAM ligands. In general, small CsPbBr_3_ QDs have high surface energies and a large number of defects, and hence they can be ripened easily, resulting in a photoluminescence (PL) redshift. Upon examining the colloidal stability of ISNCs that had been stored under an environment with relative humidity of 65% and room temperature of 25 °C, little change in emission peak and FWHM was observed over 120 days, and 90% of the initial PLQY was retained (**Figure** **3**a). By contrast, the emission wavelength of the C‐QD_113_ without Cs_4_PbBr_6_ rapidly redshifted, moving from 458 to 475 nm within 30 days, even at room temperature (Figure 3d). To compare in detail the thermal stability of the prepared QDs, they were dispersed in toluene and incubated for a period of time at 120 °C. The C‐QD_113_ were easily ripened and fused together at high temperatures, and the emission gradually shifted to longer wavelengths, with a green emission at 500 nm observed after 60 min of incubation (Figure 3e). The XRD data showed that a nonluminescent CsPb_2_Br_5_ tetragonal phase was formed simultaneously (Figure 3f). In the case of the ISNCs, the intensity of the emission centered at ≈510 nm increased slightly with time during the annealing process, but there was no shift in the position of the emission peak maximum and the blue emission was maintained with a slight decrease in PL intensity (Figure 3b). Furthermore, other crystal structures such as CsPb_2_Br_5_ were not generated, and the diffraction patterns indicated that the crystallinity of the ISNCs was improved (Figure 3c). These results clearly demonstrated that the Cs_4_PbBr_6_ effectively suppressed heat‐induced aggregation and decomposition of small CsPbBr_3_ QDs. These significant changes could be attributed to a reduction in surface energy via surface passivation. When comparing the water contact angles of ISNC and C‐QD_113_ films, the contact angle of the ISNC film was 107.2°, i.e., greater than that of the C‐QD_113_ film, which was 65.4° (Figure S4, Supporting Information). The increased contact angle indicates a reduction in surface energy due to effective passivation of unsaturated atoms by hydrophobic ligands.^[^ ^32^, ^34^ ^]^

### 2.3 Effects of Cs_4_PbBr_6_ NCs on CsPbBr_3_ QDs Quality

We hypothesized that the cause of the high PLQY and good thermal stability of the ISNCs was related to the presence of the Cs_4_PbBr_6_ NCs generated with the CsPbBr_3_ QDs. To investigate the role of the Cs_4_PbBr_6_ NCs, we conducted a systematic study to monitor the differences in the optical properties, defect levels, and morphology of CsPbBr_3_ QDs with different amounts of added Cs_4_PbBr_6_ NCs. For this purpose, nonluminescent pure Cs_4_PbBr_6_ NCs (denoted by NC_416_) with diameters of 13.6 nm were prepared separately via a previously reported method (Figure S5, Supporting Information).^[^ ^49^ ^]^ CsPbBr_3_ QDs were separated from a crude solution and then mixed with Cs_4_PbBr_6_ NCs in different weight ratios from 0 to 5 in a nonpolar solvent. The separation of the CsPbBr_3_ QDs from the crude solution involved extraction via a size selection process using methyl acetate as an antisolvent; a clear emission spectrum peaking at 477 nm was observed for the separated CsPbBr_3_ QDs. Because Cs_4_PbBr_6_ NCs and relatively large CsPbBr_3_ QDs were removed during the separation process, the absorption peak at 313 nm disappeared, and the PL peak was slightly blueshifted. The XRD and TEM results for the CsPbBr_3_ QD sample verified that the rhombohedral Cs_4_PbBr_6_ phase was not present and that the sample consisted entirely of CsPbBr_3_ QDs of 4.3 nm in size (Figure S6, Supporting Information).

**Figure 4:**
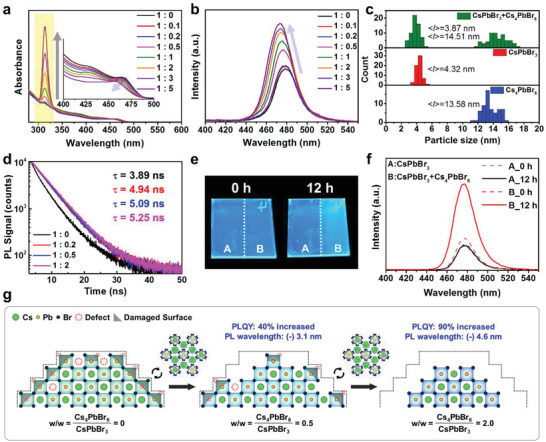
Characterization of the interaction between CsPbBr_3_ QDs and Cs_4_PbBr_6_ NCs. a) UV–vis absorption and b) PL spectra for various ratios of separately prepared CsPbBr_3_ QDs (S‐QD_113_) and Cs_4_PbBr_6_ NCs (NC_416_). c) NC size histograms before and after mixing S‐QD_113_ and NC_416_ at a weight ratio of 1:2. d) PL decay curves of S‐QD_113_ and mixed S‐QD_113_ and NC_416_. e) Photographs acquired under UV light illumination of the as‐prepared sample (left) and the sample after storage for 12 h under ambient conditions (right). f) PL spectra of film in part A and B of the sample slide after 0 and 12 h. Part A of the film was coated with S‐QD_113_ only and part B was sequentially coated with NC_416_ and then S‐QD_113_. g) Schematics of S‐QD_113_ surfaces produced by mixing S‐QD_113_ and NC_416_ in weight ratios from 1:0 to 1:2. Imperfect octahedrons on the S‐QD_113_ surface were peeled off upon addition of NC_416_.

After mixing the separated CsPbBr_3_ QDs (denoted by S‐QD_113_) and NC_416_ for 1 h at room temperature in a weight ratio of 1:0 to 1:5, changes in morphology and optical properties were observed in a nonpolar solvent (**Figure** **4**). As the relative NC_416_ amount was increased, the absorption intensity at 313 nm increased, while the first exciton peaks of S‐QD_113_ gradually decreased in intensity and were blueshifted (Figure 4a). The emission peaks were also shifted to shorter wavelength, and the PL intensity was concomitantly improved (Figure 4b and Figure S7 (Supporting Information)). In addition to the optical properties, changes in particle size and morphology were observed. In the 1:2 ratio mixed solution, the size of the S‐QD_113_ decreased from 4.32 to 3.87 nm, while the NC_416_ size increased from 13.38 to 14.51 nm (Figure 4c and Figure S8 (Supporting Information)). The morphology of the NC_416_ transformed from hexagonal to truncated diamond and assembled into zigzag shapes (Figure S9, Supporting Information). The etching of S‐QD_113_ and the variation in the NC_416_ shape are a result of the high surface energy of the small CsPbBr_3_ QDs and the structural lability of perovskite as a function of its ligand environment. The CsPbBr_3_ phase was successfully converted into the Cs_4_PbBr_6_ phase and vice versa using excess OLAM and OA in a previous study.^[^ ^50^ ^]^ In our system, NC_416_ had a relative excess of the OLAM ligand, so small S‐QD_113_ were easily etched and the NC_416_ size was increased (Figure S10, Supporting Information).

Time resolved photoluminescence (TRPL) measurements were performed to determine the effects of the PLQY enhancement along with NC etching on the optical spectroscopic characteristics (Figure 4d). Decay curves were used to analyze the excited state radiative relaxation dynamics. The S‐QD_113_ decay curve was fitted to a biexponential function with a 3.89 ns average lifetime. When S‐QD_113_ was mixed with NC_416_, the decay curve of the resultant mixture fitted a monoexponential function well, and the average lifetime was gradually increased to 5.25 ns. This increment in the average lifetime could be a result of various effects, such as energy transfer between S‐QD_113_ and NC_416_, a S‐QD_113_ size effect, and a reduction in trap‐state density; hence, we decided to examine each of these possible causes in turn. First, Cs_4_PbBr_6_ NCs have a wider bandgap than CsPbBr_3_ NCs, so energy transfer is possible when the distance between the two materials is sufficiently close. Xuan et al. reported that the lifetime and PLQY were increased in a perovskite composite, in a study in which CsPbBr_3_ NCs were embedded in Cs_4_PbBr_6_ NCs.^[^ ^38^ ^]^ In addition, Chen et al. synthesized CsPbBr_3_‐embedded Cs_4_PbBr_6_ and analyzed the effect of metal halide interlayer in determining their photoluminescence excitation (PLE) properties.^[^ ^51^ ^]^ To investigate the energy transfer, we acquired PLE and PL spectra of S‐QD_113_ and ISNCs for various the excitation wavelengths (Figure S11, Supporting Information). The S‐QD_113_ emission intensity gradually decreased as the excitation wavelength increased, in line with the trend observed for the PLE spectrum. The PLE spectrum of the ISNCs included a sharp drop centered at around 313 nm corresponding to the Cs_4_PbBr_6_ NC absorption peak. These results demonstrate that the origin of the PL of the ISNCs can be attributed to band edge emission of the CsPbBr_3_ QDs rather than energy transfer from the Cs_4_PbBr_6_ NCs. In the latter case, the ISNC emission would have improved significantly before the Cs_4_PbBr_6_ NC absorption region. Therefore, we excluded the energy transfer effect in ISNCs. Second, for perovskites, lifetimes tend to decrease as bandgaps widen.^[^ ^52^, ^53^, ^54^, ^55^ ^]^ Since S‐QD_113_ is in the strong quantum confinement regime, the lifetime is expected to decrease with the decrease in particle size. However, in our study, when NC_416_ was mixed with S‐QD_113_, the size of the S‐QD_113_ particles decreased slightly because of surface etching. This result, indicating a longer lifetime for smaller NCs, was contrary to our expectation. Therefore, we conclude that surface passivation effects are likely to be the main reasons for the longer lifetime, as reported previously.^[^ ^56^, ^57^ ^]^ The Cs_4_PbBr_6_ NCs might promote the elimination of defects and radiative recombination in the CsPbBr_3_ QDs.

To investigate in detail the optical properties of the NCs, the temperature‐dependent PL of the S‐QD_113_ and ISNC samples was measured (Figure S12, Supporting Information). The PL intensity of the S‐QD_113_ sample gradually decreased as the temperature increased, in agreement with previous reports.^[^ ^58^, ^59^, ^60^ ^]^ In metal halide perovskites, because of the low exciton binding energy, excitons are dissociated into free charge carriers by thermal energy, promoting nonradiative decay.^[^ ^60^, ^61^, ^62^ ^]^ However, the ISNCs displayed a constant PL intensity, for the entire temperature range from 20 to 300 K. As the exciton binding energies of NCs are highly dependent on the size of the NCs,^[^ ^59^, ^62^ ^]^ we expect that the exciton binding energy of the S‐QD_113_ (≈4.32 nm) and ISNC (≈4.33 nm) samples would be similar. Therefore, the constant ISNC PL intensity as a function of temperature suggests that nonradiative decay paths are substantially reduced by the presence of Cs_4_PbBr_6_.

In addition, we induced an interaction between the S‐QD_113_ and NC_416_ samples on the substrate to identify the effects of defect passivation in the solid state. First, half of the substrate was covered with Kapton tape (3M #5413) and then NC_416_ was spin coated on the exposed part. Subsequently, the experiment proceeded in order with the removal of ligands with methyl acetate, peeling off the tape, and coating S‐QD_113_ over the entire substrate. As a result, half of the substrate was covered with only S‐QD_113_, and the other coated with a double layer, with S‐QD_113_ and NC_416_ in contact at the layer interface (Figure S13, Supporting Information). For this as‐prepared film, initially there was no difference in emission between the two regions (A and B); however, the PL intensity was greatly improved in the double layer (layer B) after 12 h (Figure 4e,f). Although the interaction was relatively slow in the solid state, the S‐QD_113_ defects were also passivated similar to the solution. We summarized the above‐described mechanistic findings in a schematic illustration (Figure 4g). Surface reconstruction takes place at the interfaces between the CsPbBr_3_ QDs and Cs_4_PbBr_6_ NCs, and imperfect octahedrons on the CsPbBr_3_ QD surfaces are reduced during the etching process. In this process, the CsPbBr_3_ QD crystal size decreases, resulting in a blueshift of the emission wavelength, and the optical properties and colloidal stability are increased by the addition of Cs_4_PbBr_6_ NCs.

### 2.4 Fabrication and Characterization of Blue Perovskite LEDs

**Figure 5:**
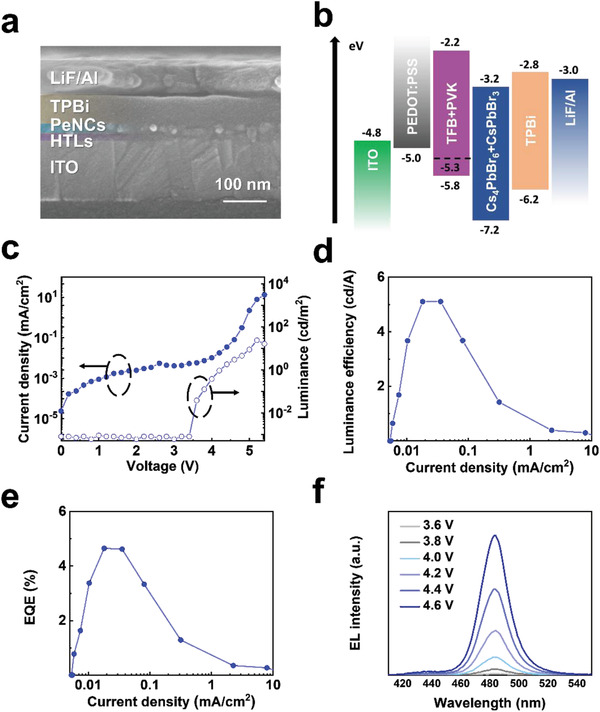
Blue perovskite LED device structure and performance characteristics. a) Cross‐sectional SEM image of ISNCs LED. b) Energy band diagram of the materials employed in LEDs. c) Current density–voltage (*J*–*V*) curve and luminance–voltage (*L*–*V*) curve, d) luminous efficiency–current density (LE–*J*) curve, and e) EQE–current density (EQE–*J*) curve of ISNCs LEDs. f) EL spectra of ISNCs LEDs at various voltages.

Encouraged by the high PLQY and stability of the ISNCs, we constructed LEDs with glass/indium tin oxide (ITO)/poly(3,4‐ethylenedioxythiophene):poly(*p*‐styrene sulfonate) (PEDOT:PSS)/poly[(9,9‐dioctylfluorenyl‐2,7‐diyl)‐*co*‐(4,4ʺ‐(*N*‐(4‐sec‐butylphenyl)diphenylamine)] (TFB)+ poly(9‐vinylcarbazole) (PVK) (30 nm)/ISNCs (20 nm)/2,2ʺ,2ʺ‐(1,3,5‐benzinetriyl)‐tris(1‐phenyl‐1‐*H*‐benzimidazole) (TPBi) (70 nm)/LiF (1 nm)/Al (100 nm) structures. The thickness of each layer was confirmed via the acquisition of cross‐sectional scanning electron microscopy (SEM) images (**Figure** **5**a). Energy band diagrams for the materials employed in the LEDs are shown in Figure 5b. Due to the low Cs_4_PbBr_6_ HOMO energy level (7.2 eV), a triple hole injection layer including a polymer with a deep work function was used for efficient hole injection. The device performance characteristics of the ISNCs LEDs are shown in Figure 5c–f and Table S1 (Supporting Information). The LEDs fabricated with ISNCs exhibited a maximum luminance of 23 cd m^−2^ and EQE of 4.65% at wavelength of 480 nm. Histogram of maximum EQEs of ISNCs LEDs are shown in Figure S14 (Supporting Information).

One of the most important problems for blue‐emitting metal halide perovskites is the spectral instability that results from halide segregation. LED emission spectra measured using various voltage bias values are shown in Figure 5e. Because the ISNCs exhibited blue emission at 480 nm without halide mixing, the ISNC LEDs exhibited stable electroluminescence (EL) emission spectra over the entire range of device operating voltages.

## 3 Conclusion

In summary, we demonstrated a novel synthetic method to simultaneously obtain ultrasmall CsPbBr_3_ QDs and Cs_4_PbBr_6_ NCs by controlling the ratio of the Cs and Pb precursors as well as the reaction temperature. CsPbBr_3_ QDs of 3.5 and 4.3 nm in size – smaller than the exciton Bohr radius – were synthesized at 60 and 80 °C, respectively. These QDs emitted blue light at 470 and 477 nm, respectively, with high PLQYs of more than 90%. The Cs_4_PbBr_6_ NCs eliminated the imperfect octahedron defect sites on the surfaces of the CsPbBr_3_ QDs, which resulted in suppression of nonradiative recombination; this was confirmed via TRPL and temperature‐dependent PL measurements. In addition, the colloidal and thermal stabilities of the ISNCs were significantly enhanced by suppressing particle aggregation and fusion. We realized efficient blue LEDs with a maximum EQE of 4.65% at 480 nm. In particular, the EL spectra were stable across various applied voltages, without exhibiting any peak shifts. This surface defect etching method, which relied on CsPbBr_3_ and Cs_4_PbBr_6_ phase surface reconstruction, was found to be effective even for solid‐state films. Thus, our strategy could expand the development of a wide range of perovskite optoelectronic applications, including solar cells and NC‐based LEDs, thanks to the introduction of a new method for controlling the amount of defects.

## 4 Experimental Section

### Materials

OLAM (98%), HBr (48%), Cs_2_CO_3_ (99.9%), PbO (99.999%), ODE (90%), OA (90%), and lead bromide (PbBr_2_, 98%) were purchased from Sigma‐Aldrich. Acetonitrile (ACN, 99.5%), toluene (99.8%), methyl acetate (99.5%), and hexane (99.5%) were purchased from Samchun Chemicals. All the chemicals were used without further purification.

### Preparation of OLAM‐Br Precursor

The OLAM‐Br salt was synthesized according to a previous method with some modifications.^[^ ^41^ ^]^ In a typical synthesis, OLAM (10 mL) and HBr solution (1.3 mL) were loaded into a 50 mL three‐necked round‐bottom flask, and the resulting solution was stirred under an Ar atmosphere at 120 °C for 2 h. Then, it was heated to 150 °C and left to react for an additional 30 min. After cooling the solution to 100 °C, it was vacuum dried for 1 h to remove any residual water. The precursor was collected in an Ar‐filled vial and stored in a glove box for further use.

### Synthesis of ISNCs

Mixed CsPbBr_3_–Cs_4_PbBr_6_ NCs were prepared via an OLAM‐Br precursor hot‐injection method. Typically, Cs_2_CO_3_ (32.6 mg, 0.1 mmol), PbO (44.6 mg, 0.2 mmol), OA (1.0 mL), and ODE (10 mL) were stirred in a 50 mL three‐necked round‐bottom flask and degassed under vacuum at 120 °C for 1 h. After complete solubilization of the reaction mixture, the flask was filled with Ar and heated (or cooled) to obtain the desired temperature (60–160 °C). Then, the preheated OLAM‐Br solution (0.9 mL) was swiftly injected into the reaction mixture. The reaction was quenched in an ice water bath after 30 min.

### Purification of Synthesized NCs

The crude solution was transferred to a 50 mL conical tube and ACN and toluene were then added to the solution in a volume ratio of 1:2:3 (crude mixture:ACN:toluene). The nanocrystals were precipitated in a centrifuge at 7000 rpm for 5 min. The supernatant was discarded, and the precipitate was collected and dissolved in hexane. One more centrifugation (7800 rpm, 5 min) was required to purify the NCs and obtain the final product. The clear supernatant was collected and used for future studies.

To eliminate the Cs_4_PbBr_6_ NCs from the crude solution, methyl acetate was used as an antisolvent instead of the mixture of ACN and toluene. Typically, methyl acetate (5 mL) was added to the crude solution (5 mL). This solution was centrifuged at 7800 rpm for 5 min and the precipitate was discarded. An additional methyl acetate (20 mL) was added to the supernatant, and this mixture was centrifuged at 7800 rpm for 5 min. The precipitated CsPbBr_3_ QDs (S‐QD_113_) were used in further studies after dispersion in hexane.

### Synthesis of Pure NC_416_

Monodisperse Cs_4_PbBr_6_ NCs were synthesized according to a previously reported method.^[^ ^49^ ^]^ The Cs‐oleate precursor and NCs were prepared in air. For the Cs‐oleate preparation, Cs_2_CO_3_ (0.4 g) and OA (8 mL) were loaded in a 20 mL vial and stirred on a hot plate at 150 °C for 20 min. In a typical synthesis, PbBr_2_ (36.7 mg), OA (0.2 mL), OLAM (1.5 mL), and ODE (5 mL) were stirred at 150 °C until the solution became transparent. After cooling the solution to 80 °C, preheated Cs‐oleate (0.75 mL) was swiftly injected into it. The reaction was quenched in an ice water bath after 3 min. The crude solution was washed via centrifugation (4500 rpm, 10 min), which was followed by redispersion in hexane.

### Synthesis of C‐QD_113_

Small CsPbBr_3_ QDs were synthesized according to a previously reported method with some modifications.^[^ ^48^ ^]^ The synthetic approach was based on hot injection of the Cs‐oleate precursor. In brief, PbBr_2_ (69 mg) and ODE (5 mL) were loaded in a 50 mL three‐necked round‐bottom flask and degassed under vacuum at 120 °C for 1 h. Dried OA (0.6 mL) and OLAM (0.3 mL) were injected to the reaction mixture at 120 °C under Ar. After complete solubilization of the reaction mixture, preheated Cs‐oleate precursor (0.4 mL) was swiftly injected into the reaction mixture at 90 °C. The reaction was quenched in an ice water bath after 10 s. For Cs‐oleate preparation, Cs_2_CO_3_ (0.4 g), OA (1.2 mL), and ODE (15 mL) were stirred in a 50 mL three‐necked round‐bottom flask and degassed under vacuum at 120 °C for 1 h. Then, the solution was heated to 150 °C and reacted for an additional 30 min. The Cs‐oleate precursor was preheated to 100 °C before use. Purification of the obtained QDs was achieved via the method mentioned above.

### Preparation of Ex Situ Mixed S‐QD_113_ and NC_416_

The solution was prepared by adding an amount of NC_416_ to S‐QD_113_ (2 mg) to achieve the desired weight ratio in hexane (500 µL). After the addition of NC_416_, it was observed that the PL intensity increased within a few seconds. To achieve a homogeneity, the mixture was stirred for 1 h at room temperature. It is worth noting that when the QD_113_:NC_416_ weight ratio exceeded 1:5, the QD_113_ sample was completely etched and the emission was lost. To apply this system to the solid state, glass substrates, Kapton tape, QD_113_ (10 mg mL^−1^ in hexane), and NC_416_ (20 mg mL^−1^ in hexane) were used. The glass substrates were first cleaned by sonification while sequentially immersed in deionized water, acetone, and isopropyl alcohol. Then, half of the glass was covered with Kapton tape, and NC_416_ was spin coated at 3000 rpm for 1 min. To avoid dissolution of NC_416_ layer, methyl acetate was then spin coated on the slide twice at 3000 rpm for 1 min. After peeling off the Kapton tape, S‐QD_113_ was spin coated at 3000 rpm for 1 min on the entire substrate.

### Thermal Stability Test

After dissolving the NCs in toluene and adjusting the concentration to 10 mg mL^−1^, a change in the PL spectrum of the sample in a 120 °C oil bath was observed as the time elapsed.

### Device Fabrication

ITO‐patterned glass substrates were cleaned by sonification while sequentially immersed in deionized water, acetone, and isopropyl alcohol. The PEDOT:PSS layer was spin coated at 5000 rpm for 40 s on the ITO substrates after 30 min of UV treatment. The slide was then transferred into a glove box and annealed at 140 °C for 10 min. TFB and PVK (volume ratio 1:1) were blended and dissolved in chlorobenzene such that the concentration of the mixture was 3 mg mL^−1^. The TFB/PVK mixture solution was spin coated on the substrates (3000 rpm, 40 s) and then annealed at 130 °C for 20 min. Perovskite NCs were then spin coated on the substrates (2000 rpm, 30 s). Finally, the slide was sequentially coated with TPBi (50 nm), LiF (1 nm), and Al (100 nm) by thermal evaporation.

### Characterization

Absorption spectra were acquired by a Shimadzu UV‐1800 UV–vis spectrometer. PL spectroscopy was carried out and quantum yields were obtained for the NCs via the use of a quantum efficiency measurement system (Otsuka QE‐2000). Photoluminescence emission spectra were obtained by using an Agilent fluorescence spectrophotometer. XRD was performed by using a Rigaku Ultimate‐IV X‐ray diffractometer operated at 40 kV and 200 mA using the Cu K*α* line (*λ* = 1.5418 A). TEM images were acquired by a JEOL JEM‐2100 microscope with an acceleration voltage of 200 kV using copper grids (Ted Pella, USA). The particle sizes and distributions were measured using DigitalMicrograph software in TEM images. TRPL spectra were obtained by means of a time‐correlated single‐photon counting setup (FluoTime 300, PicoQuant) at room temperature. ^1^H nuclear magnetic resonance spectra were acquired using a Bruker AVANCE III HD (400 MHz) spectrometer. The residual proton signal of the deuterated solvent was selected as the reference standard. Temperature‐dependent PL measurements were performed in the temperature range of 20–300 K using a liquid helium cooler. PL spectra of the nanocrystal films were obtained using the Agilent fluorescence spectrophotometer. Water contact angles were measured using a drop shape analyzer (DSA‐100, Krüss). Cross‐sectional SEM images of the device structures were obtained using a Nova Nano230 FEI SEM (accelerating voltage 10 kV). To prevent the occurrence of charging, a 5 nm platinum layer was deposited on the samples via sputter coating (Emitech K575x, Tescan). The device performances of the encapsulated LEDs were measured using a Keithley 2400 sourcemeter and spectroradiometer (CS‐2000, Konica Minolta) under ambient conditions.

## Conflict of Interest

The authors declare no conflict of interest.

## Supporting information

Supporting Information

## Data Availability

Research data are not shared.
