# Supplementary material for: Highly Emissive Blue Quantum Dots with Superior Thermal Stability via In Situ Surface Reconstruction of Mixed CsPbBr3–Cs4PbBr6 Nanocrystals
Source: Adv Sci (Weinh). 2021 Dec 26;9(5):2104660. doi: 10.1002/advs.202104660 (PMC8844471; doi:10.1002/advs.202104660)
Supplement: Supplementary file 1 — Supporting Information [file ADVS-9-2104660-s001.pdf]

ADVANCED SCIENCE
Open Access

Supporting Information

for *Adv. Sci.*, DOI: 10.1002/advs.202104660

Highly emissive blue quantum dots with superior thermal stability via in situ surface reconstruction of mixed $CsPbBr_3$–Cs_4PbBr_6 nanocrystals

Hyeonjung Kim, Jong Hyun Park, Kangyong Kim, Dongryeol Lee, Myoung Hoon Song, and Jongnam Park**

Supporting Information

Highly emissive blue quantum dots with superior thermal stability via in situ surface reconstruction of mixed $CsPbBr_3$–Cs_4PbBr_6 nanocrystals

Hyeonjung Kim, Jong Hyun Park, Kangyong Kim, Dongryeol Lee, Myoung Hoon Song, and Jongnam Park**

H. Kim, K. Kim, Prof. J. Park
School of Energy and Chemical Engineering, Ulsan National Institute of Science and Technology (UNIST), UNIST-gil 50, Ulsan 44919, Republic of Korea
E-mail: jnpark@unist.ac.kr

Prof. J. Park
Department of Biomedical Engineering, Ulsan National Institute of Science and Technology (UNIST), UNIST-gil 50, Ulsan 44919, Republic of Korea

J. H. Park, D. Lee, Prof. M. H. Song
Department of Materials Science and Engineering, Ulsan National Institute of Science and Technology (UNIST), UNIST-gil 50, Ulsan 44919, Republic of Korea
E-mail: mhsong@unist.ac.kr

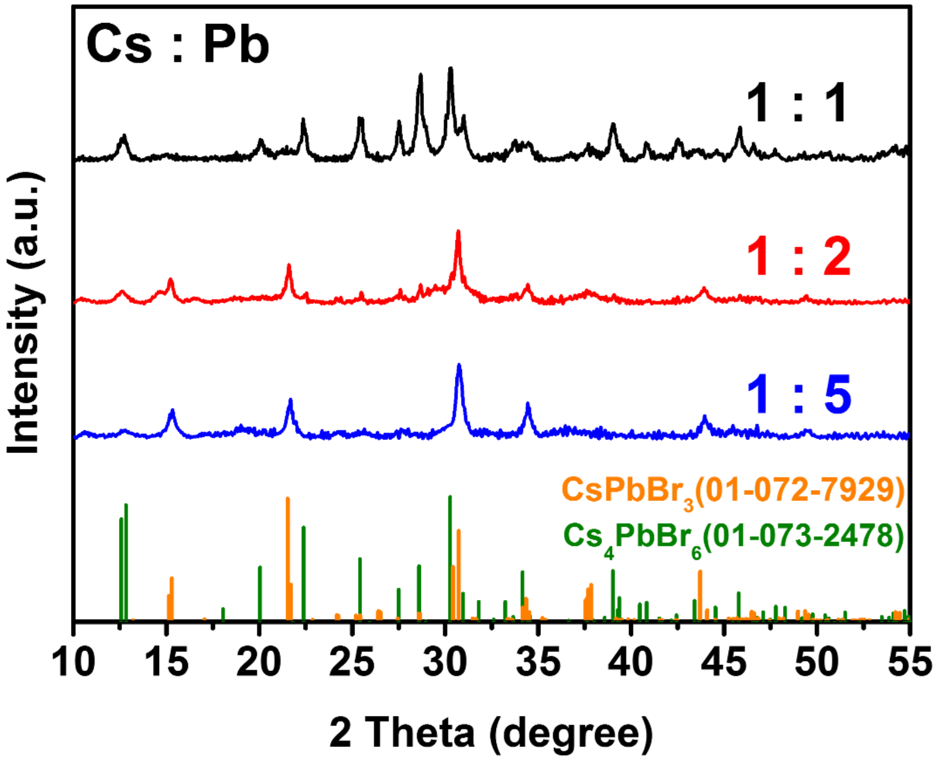

Figure S1. X-ray powder diffraction (XRD) patterns according to the feeding molar ratio of Cs and Pb precursor.

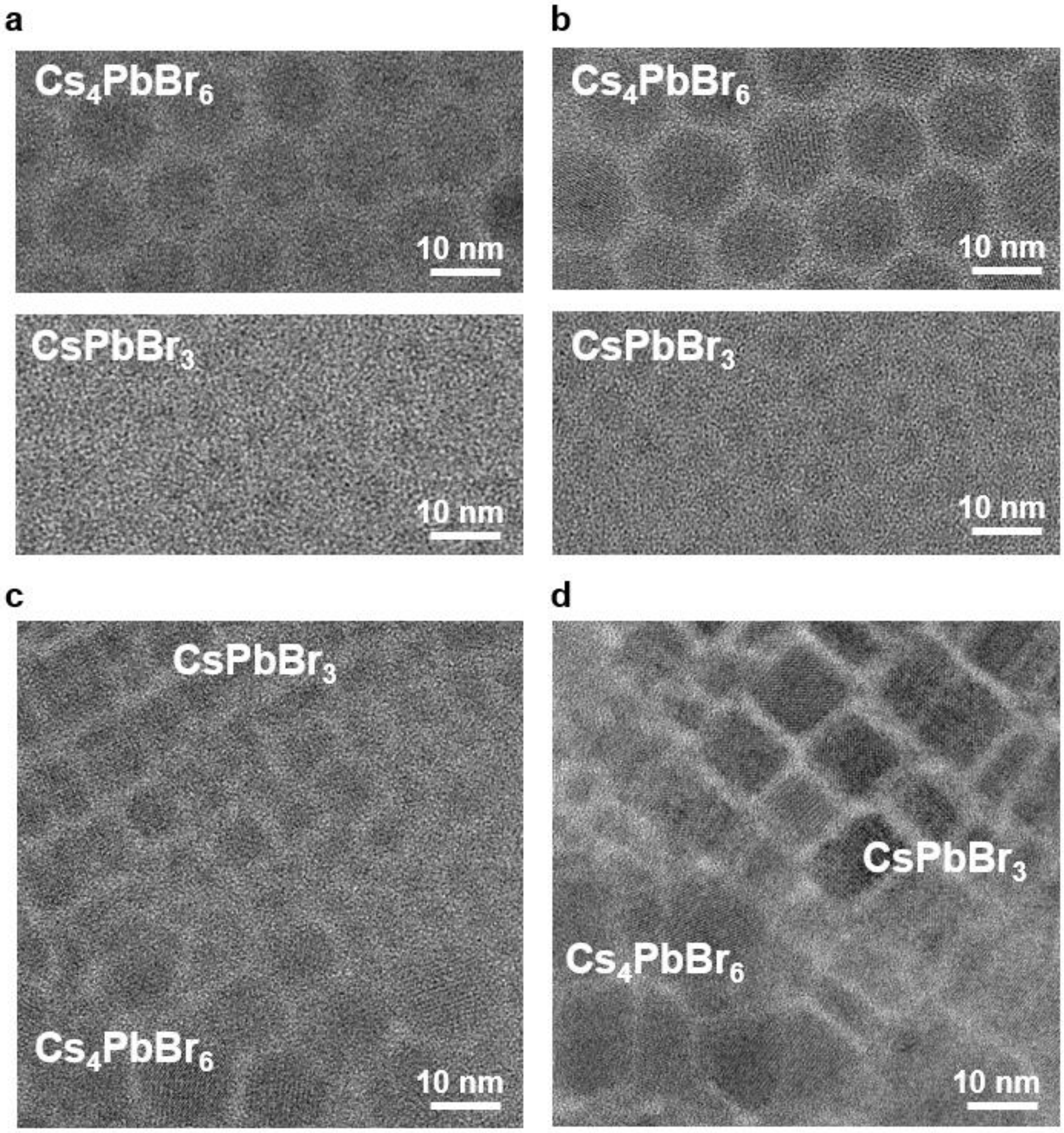

Figure S2. High magnification transmission electron microscopy (TEM) images of the in situ generated $CsPbBr_3$-Cs_4PbBr_6 nanocrystals (ISNCs) synthesized at (a) 60 ^oC, (b) 80 ^oC, (c) 120 ^oC, and (d) 160 ^oC.

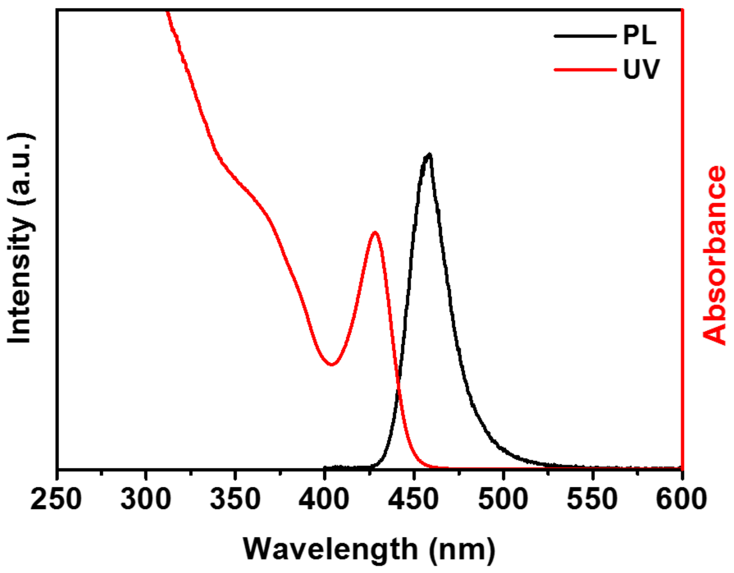

Figure S3. UV-vis absorption and PL spectra of conventional $CsPbBr_3$ quantum dots (C-QD_{113}).

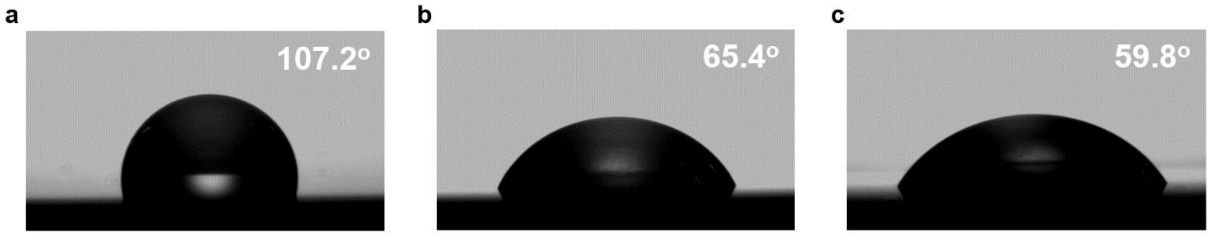

Figure S4. Water contact angles of (a) ISNCs, (b) C-QD_{113}, and (c) separated $CsPbBr_3$ QDs (S-QD_{113}) on SiO_2 wafer.

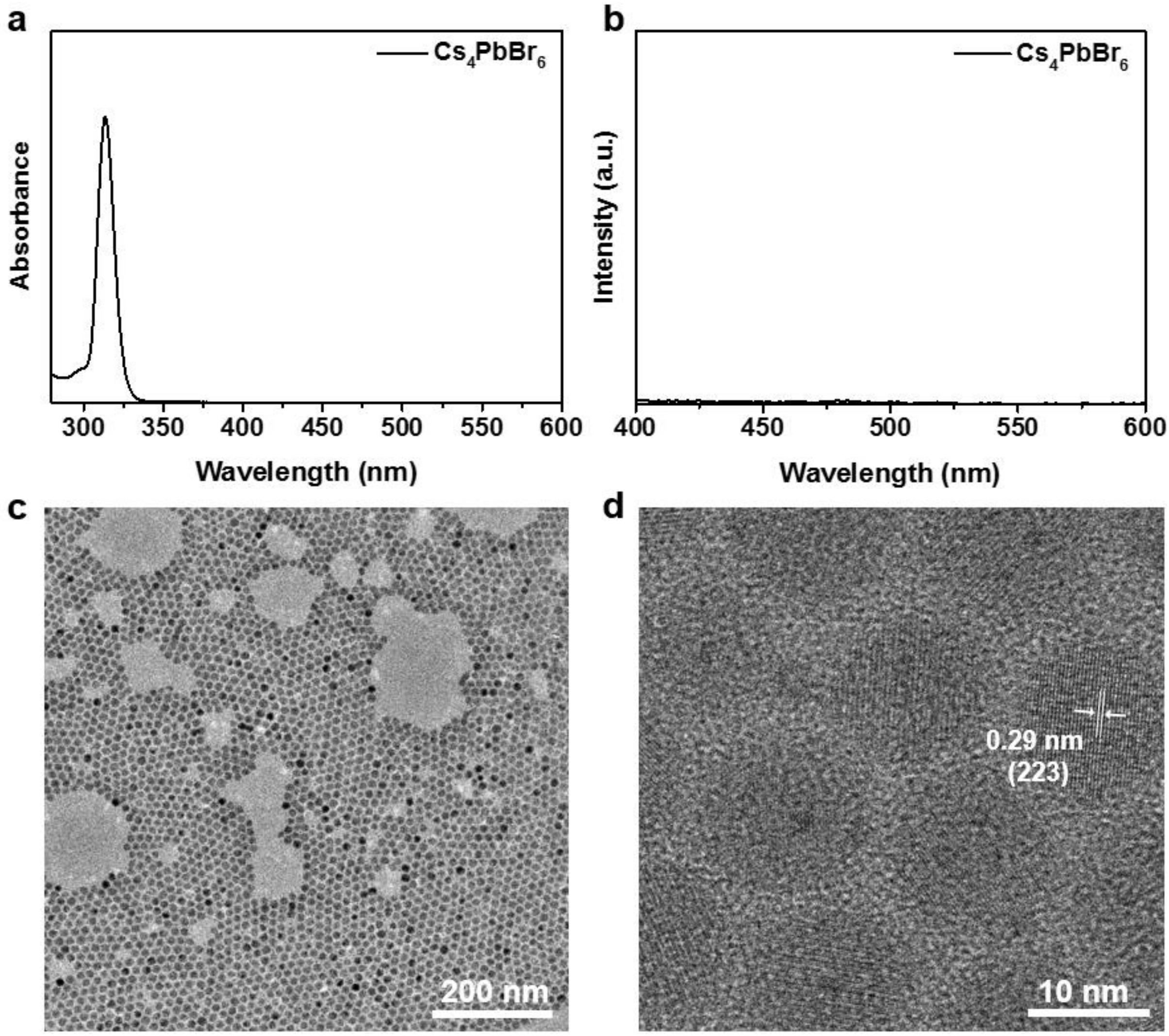

Figure S5. (a) UV-vis absorption spectrum, (b) PL spectrum, and (c,d) TEM images of pure Cs_4PbBr_6 NCs (NC_{416}).

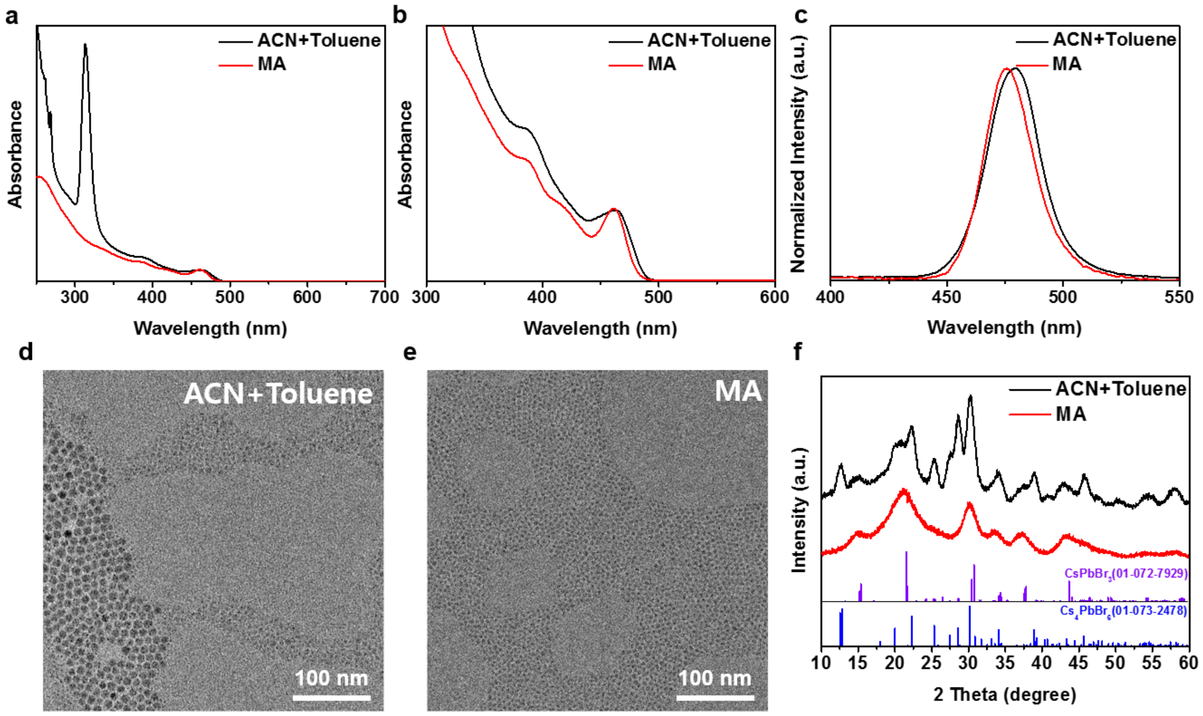

Figure S6. (a) UV-vis absorption spectra, (b) magnified view of the absorption spectra in the region 300-600 nm, and (c) Photoluminescence (PL) spectra of NCs washed with methyl acetate and the mixture of acetonitrile and toluene. TEM images of NCs washed with (d) the mixture of acetonitrile and toluene and (e) methyl acetate. (f) XRD patterns according to the washing method.

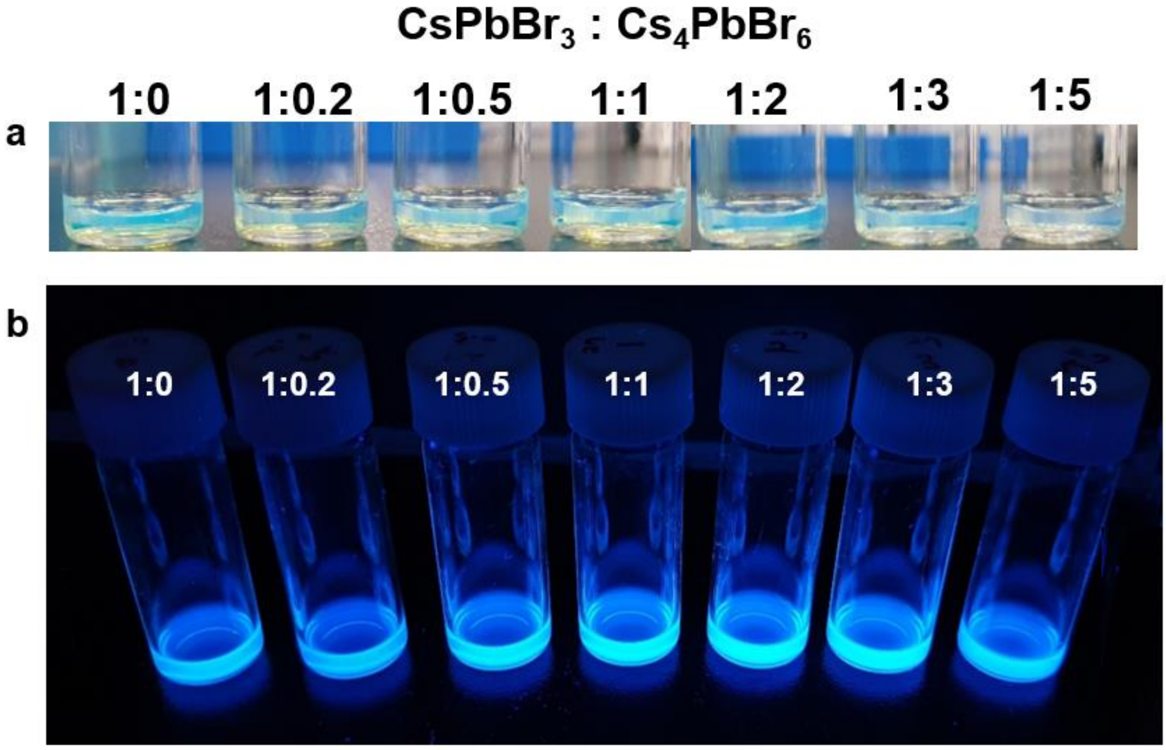

Figure S7. Photographs under (a) room light and (b) 365 nm UV light of the mixture according to the weight ratio of S-QD_{113} and NC_{416}.

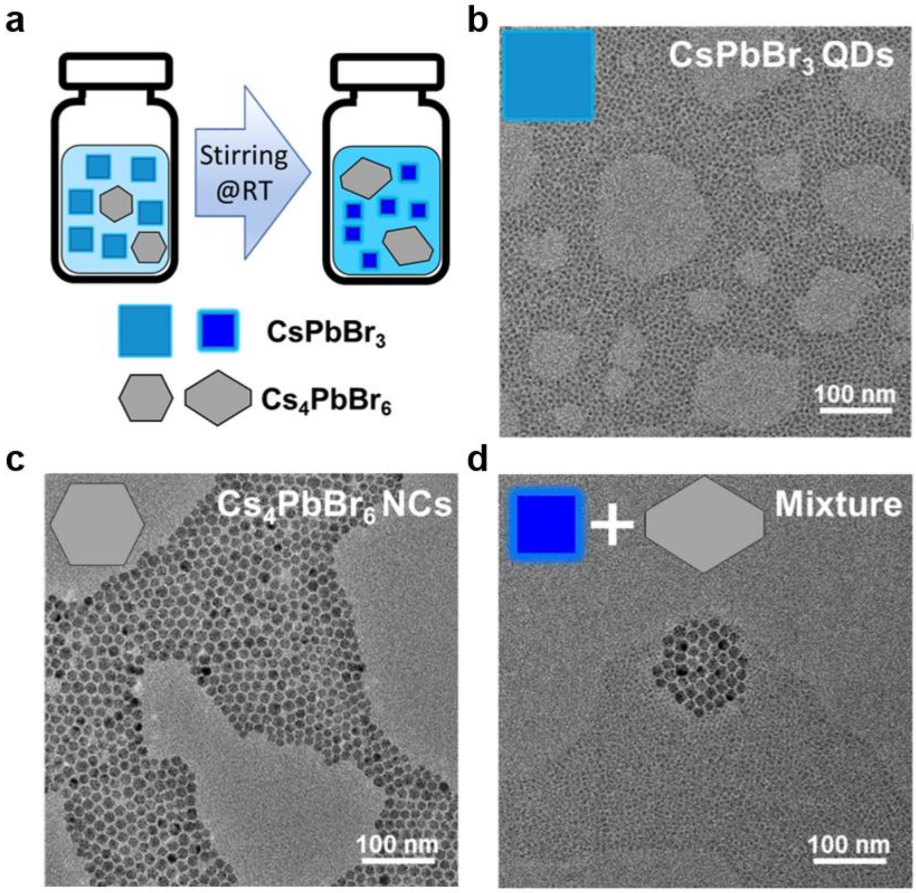

Figure S8. (a) The schematic illustration of the experiment mixing S-QD_{113} and NC_{416} at a weight ratio is 1:2. TEM images of (b) S-QD_{113}, (c) NC_{416}, and (d) mixed S-QD_{113} and NC_{416}.

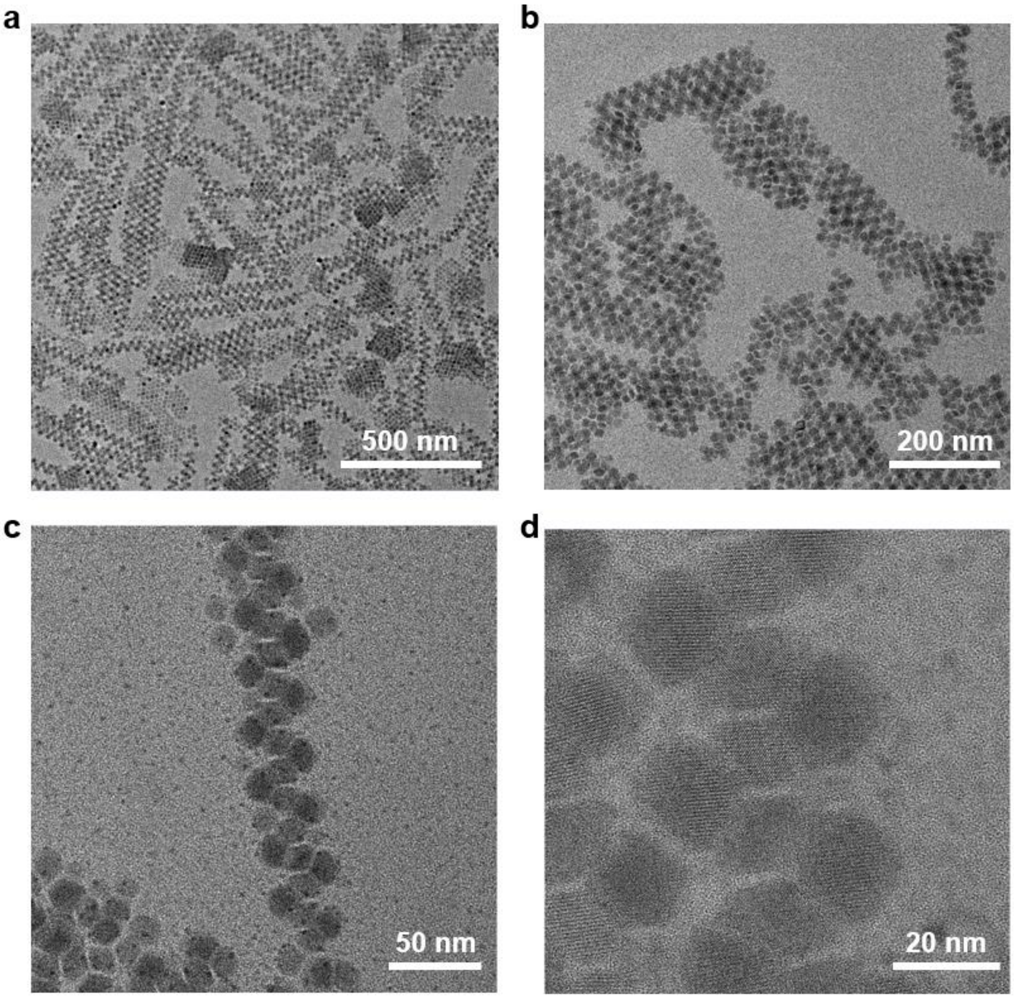

Figure S9. TEM images with different magnifications at (a) 13.5k, (b) 26.5k (c) 88k, and (d) 255k when S-QD_{113} and NC_{416} are mixed in a 1:2 weight ratio.

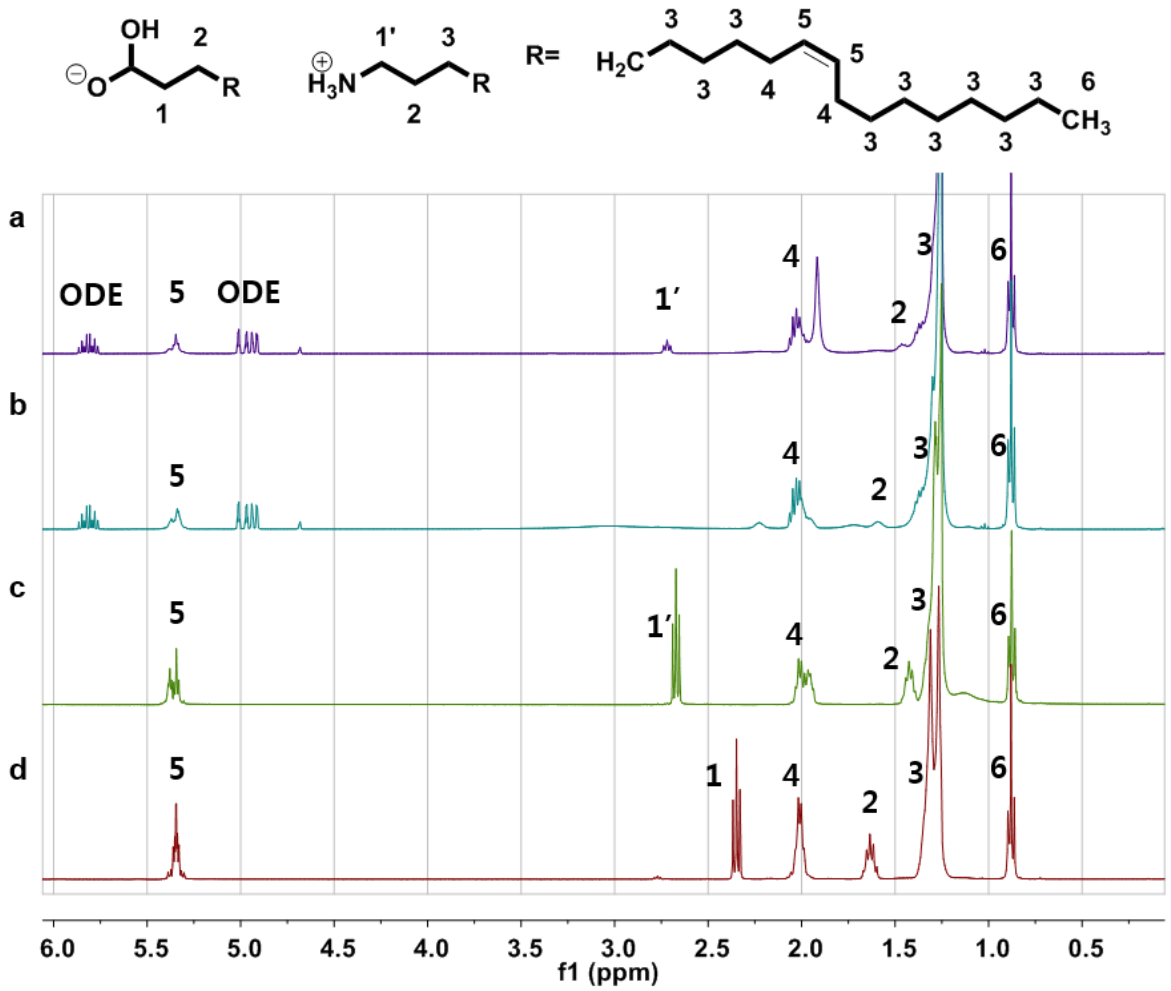

Figure S10. ^{1}H nuclear magnetic resonance spectra of (a) NC_{416}, (b) S-QD_{113}, (c) oleylamine, and (d) oleic acid dissolved in $CDCl_3$.

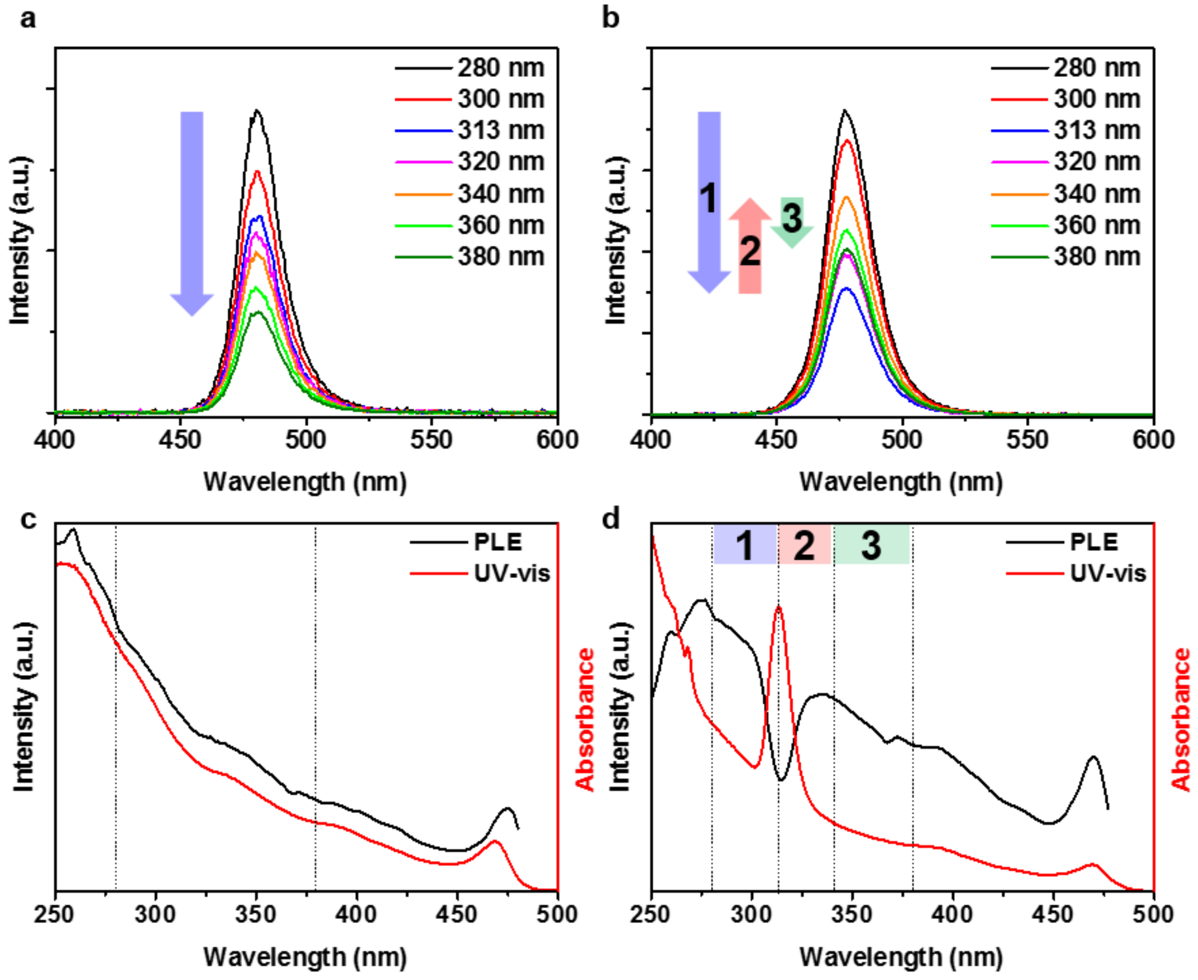

Figure S11. PL spectra of (a) S-QD$_{113}$ and (b) ISNCs as a function of excitation wavelength. UV-vis absorption and PL excitation spectra of (c) S-QD$_{113}$ and (d) ISNCs.

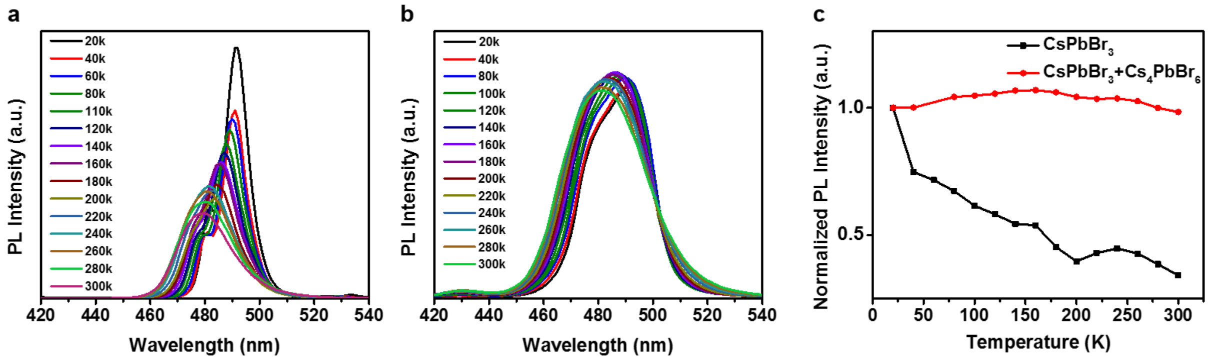

Figure S12. Temperature-dependent PL spectra of (a) S-QD$_{113}$ and (b) ISNCs in the temperature range of 20-300k. (c) Normalized PL intensities of S-QD$_{113}$ and ISNCs as a function of temperature.

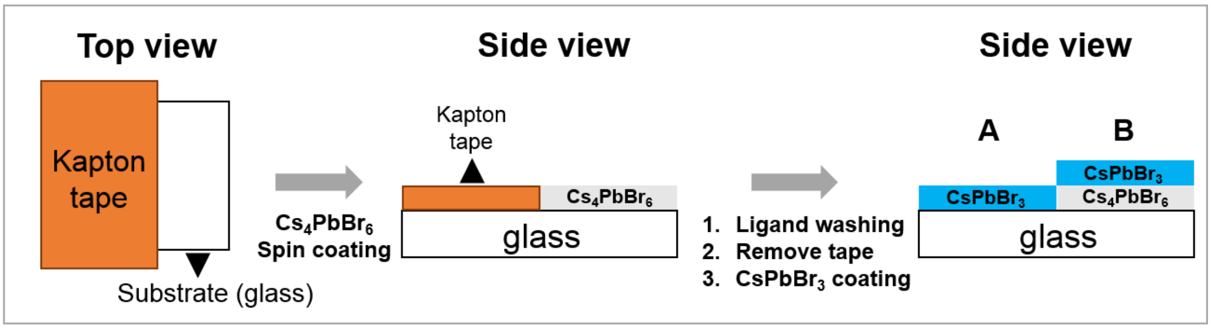

Figure S13. The schematic illustration of the mixing S-QD$_{113}$ and NC$_{416}$ in solid state.

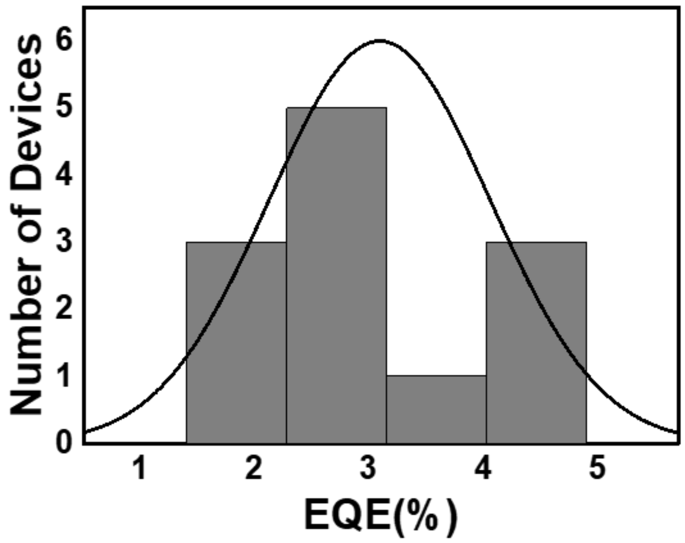

Figure S14. Histogram of maximum EQEs of ISNCs LEDs.

Table S1. Summary of device performance of ISNCs LEDs.

Conditions	*L* $_{max}$ [cd m^{-2}] @bias	LE $_{max}$ [cd A^{-1}] @bias	EQE $_{max}$ [%] @bias	EQE $_{avr}$ [%] from 12 devices
ITO/PEDOT:PSS/PVK+TFB/ISNCs/TPBi/LiF/Al	23 @ 5.2	5.10 @ 4.2	4.65 @ 4.2	3.13
